# Can pediatric rheumatologists apply available hip scoring systems in daily practice for juvenile idiopathic arthritis?

**DOI:** 10.3389/fped.2025.1436200

**Published:** 2025-03-28

**Authors:** Kaouther Maatallah, Hanene Lassoued Ferjani, Dorra Ben Nessib, Abir Dghaies, Lobna Kharrat, Fatma Majdoub, Dhia Kaffel, Wafa Hamdi

**Affiliations:** ^1^ Department of Rheumatology, Kassab Institute of Orthopedics UR17SP04, Ksar Saïd, Tunisia; ^2^ Faculty of Medicine of Tunis, Tunis El Manar University, Tunis, Tunisia

**Keywords:** juvenile idiopathic arthritis, scoring system, radiograph, children, measurement, hip

## Abstract

**Introduction:**

Scoring systems for hip involvement in juvenile idiopathic arthritis exist, however, they were not used in daily practice, and their reproducibility was not proven.

**Objectives:**

We aimed to determine the applicability, reliability, and repeatability of the two scoring systems of the hip in juvenile idiopathic arthritis patients.

**Methods:**

Two expert pediatrics rheumatologists analyzed pelvic radiographs from 25 children with JIA hip involvement. We scored the findings according to two previous valid scoring systems (The childhood Arthritis Radiographic Score of the Hip and the newly developed score proposed by the project Health-e-Child) at baseline and after three weeks. We used kappa statistics to rate inter- and intra-observer variability.

**Results:**

The joint space narrowing, erosion, and growth abnormalities had moderate to good reliability when the first score was used. However, the subchondral cysts, malalignment, and sclerosis have poor concordance in the two observers. For the second score, the intraclass correlation coefficient (ICC) was high in only one reader for head erosion (*κ* = 0.833 vs. *κ* = 0.308; *p* < 0.001), enlarged fovea (*κ* = 0.279 vs. *κ* = 0.907; *p* < 0.05), and growth abnormalities (*κ* = 0.823 vs. *κ* = 0; *p* < 0.001; *p* = 0.5). Therefore, the intra-reader agreement for head femoral measuring and centrum–column–diaphysis angle showed good reliability for only one reader. Training has only improved the observers' agreement with the assessment of growth disorders in the first score. The interpretation agreement was also increased compared to the baseline in the femoral measurements.

**Conclusion:**

The reliability of these tools seemed to be lower without electronic measurements and the pediatric rheumatologists needed more training before applying these scoring in the practice hip monitoring.

**Clinical Trial Registration:**

NCT05206968 Last Update: 01/12/2022.

## Introduction

Juvenile idiopathic arthritis (JIA) is a chronic inflammatory disease in children that affects mobility and physical function (1). It is characterized by synovial inflammation leading to joint destruction. Hip involvement occurs in 20%–50% of JIA patients and predicts poor outcomes in adulthood, leading to functional impairment and impacting the patient's quality of life (2–4). Therefore, effective management of hip involvement is crucial for disease monitoring and significantly influences treatment decisions (5, 6).

A radiographic grading system that assesses structural joint damage is essential. Such a scoring system needs to be simple to use in daily practice without requiring specialized radiology software. The Childhood Arthritis Radiographic Score of the Hip (CARSH) effectively covers osteoarticular changes in the hip joint, including space narrowing, erosions, malalignment, sclerosis, flattening of the femoral head, and growth abnormalities (7). Although the CARSH is simple and quick to use, it lacks an objective evaluation of hip modifications. Osteochondral changes in the growing hip may be limited to growth disturbances without destruction. To address this gap, the longitudinal multi-center (Health-e-Child) project developed a new scoring system for hip JIA (8). This system used the same criteria as the CARSH index and included detailed assessments of growth abnormalities such as the length and width of the femoral neck, the trochanteric-femoral head height, and the status of the physis. However, these measurements and angles were assessed using a standard electronic measurement tool, which may not be easily feasible in daily practice.

Since the validation of these scoring systems, no study has examined their reproducibility and reliability. Additionally, while these scores were developed and validated by radiologists, it is typically pediatric rheumatologists who assess structural progression in daily practice. Therefore, it is important to determine if these scores can be applied by pediatric rheumatologists. This study aimed to assess the applicability and impact of training on inter- and intra-agreement among pediatric rheumatologists in analyzing hip radiographs using the two scoring systems in JIA patients.

## Methods

### Study design

This cross-sectional observational study was conducted in the pediatric rheumatology department and included patients with JIA according to the ILAR criteria (9). We selected patients with hip involvement, defined as the presence of hip pain and/or limping, range of motion limitation, and/or abnormal findings on pelvic radiography, ultrasound, or MRI of the hip joint. All included patients had anteroposterior pelvic radiographs taken prior to the beginning of the study.

Exclusion criteria were as follows: patients for whom a new x-ray was not necessary and who did not already have pelvic x-rays on file, those with congenital hip dislocation, and patients who had undergone hip surgery.

### Reading strategy

Two experienced pediatric rheumatologists, blinded to each patient's data, independently read the pelvic radiographs of the children. Both raters underwent two training sessions prior to the assessment phase. During this time, raters received detailed scoring guidelines and sample cases not included in this study. Each rater was encouraged to discuss their grading criteria, and consensus was reached. Pathological radiographs were then interpreted twice by the same rater, at baseline and after three weeks.

**Figure 1:**
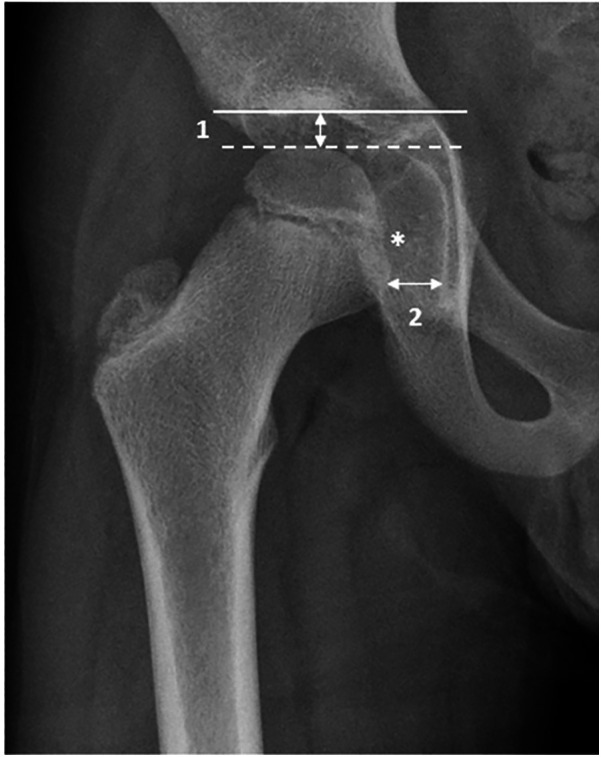
Demonstration of joint space measurement in the medial and cranial femoral head according to the study of Shelmerdine et al. (8). A solid line marks the acetabular roof, and a dashed line marks the femoral head's highest point, allowing measurement of the distance between the two lines (arrow 1). Secondly, the joint space is measured from the medial side to the femoral head's center (arrow 2) beneath the fovea (*). Reproduced with permission from “Demonstration of joint space measurements on an anteroposterior pelvic radiograph in a 9-year-old boy with juvenile idiopathic arthritis” by Susan C. Shelmerdine, Pier Luigi Di Paolo, Jasper F. M. M. Rieter, Clara Malattia, Laura Tanturri de Horatio and Karen Rosendahl, licensed under CC BY 4.0.

Two scoring systems were used: the CARSH score (7) and a newly developed score (the new scoring system of hip JIA) (8) (Figure 1). The CARSH score assessed the following radiographic abnormalities: joint space narrowing (JSN), erosion, growth abnormalities, subchondral cysts, malalignment, sclerosis of the acetabulum, and avascular necrosis of the femoral head. Each hip was assigned a score ranging from 0 to 16, with 0 indicating no abnormalities. For each abnormality, a score of 0 was given if the abnormality was absent. If abnormalities were present, JSN was scored from 1 to 3 based on severity, erosion from 1 to 4, and growth abnormalities, subchondral cysts, and malalignment were scored as 1 or 2. Sclerosis of the acetabulum was scored as 1, and avascular necrosis of the femoral head, due to its greater severity, was scored as 2. The new scoring system of hip JIA evaluated destructive changes and growth abnormalities. The first part included the following features: bone erosions in three locations (femoral head, acetabulum, and femoral neck), flattening of the femoral head using the Mose circle, enlargement of the fovea (scored from 0 to 2), presence of sclerosis in two locations (femoral head and acetabulum), and the height of the joint space (in medial and cranial locations).

The second part of the new scoring system of hip JIA measured the length and width of the femoral neck, the trochanteric-femoral head height, and the centrum–collum–diaphysis angle.

### Data collection

Overall demographic data, disease characteristics (symptom duration, disease presentation, JIA subtypes, HLA-B27 positivity), disease activity (Juvenile Arthritis Disease Activity Score, JADAS10 (10), and treatment were collected.

### Ethical statement

Parental or legal guardian consent was required before patient inclusion. The study was approved by the local institute's ethics committee.

### Statistical analysis

Intra-observer and inter-observer reliability were determined using Cohen's Kappa (*κ*) values for nominal qualitative variables and intra-class correlation (ICC) for quantitative ordinal variables. The kappa values and ICC were interpreted using the Landis and Koch guidelines (11), where *κ* or ICC >0.6 is good agreement, 0.41–0.60 is moderate, 0.21–0.40 is slight, 0.00–0.20 is poor and <0.00 is absent.

## Results

Twenty-five patients were included in the study. Demographic and disease characteristics are summarized in Table 1. The JIA subtypes observed were as follows: Enthesitis-related arthritis (16 patients), psoriatic arthritis (3 patients), oligoarthritis (4 patients), polyarticular with rheumatoid factor (1 patient), and polyarticular without rheumatoid factor (1 patient).

**Table 1:** Patients’ characteristics.

Characteristics	Data
Total number, (*n*)	25
Age, mean ± S.D, years, [range]	13.9 ± 4.3 [5–22]
Male, *n* (%)	15 (60)
BMI, mean ± S.D, Kg/m^2^	18.3 ± 5 [13–22]
Age of onset, mean ± S.D, years, [range]	10.5 ± 3.2 [4–16]
Age at diagnosis, mean ± S.D, years, [range]	11.5 ± 3.8 [4–19]
Symptom duration, months ± S.D	43 ± 32 [4–156]
Antigen (HLA) B27 positivity, *n*	2
Extra-articular manifestations, (*n*)	Uveitis (1), psoriasis (1)
JADAS, mean ± S.D, [range]	6.7 ± 6.3 [0–18.5]
Treatments, (*n*)	
NSAIDs	18
Methotrexate	18
bDMARD	3

S.D, standard deviation; *n*, number; BMI, body mass index; JADAS, juvenile arthritis disease activity; HLA, human leucocyte antigen; NSAIDs, non-steroidal anti-inflammatory drugs; bDMARD, biological disease modifying anti-rheumatic drugs.

**Figure 2:**
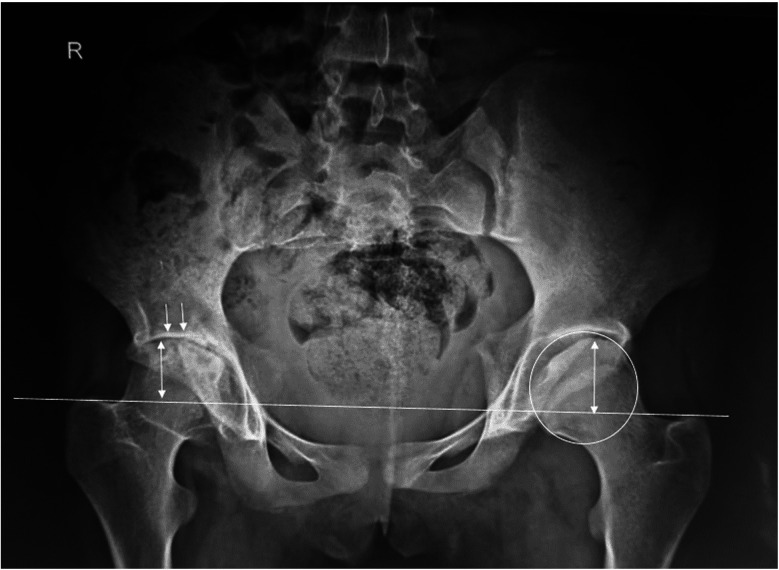
Bilateral hip radiograph of an 18-year-old girl with polyarticular JIA of 10 years duration. The radiograph showed bilateral joint space narrowing (white arrows), femoral head flatting (white circle), and asymmetric trochanteric femoral height.

**Figure 3:**
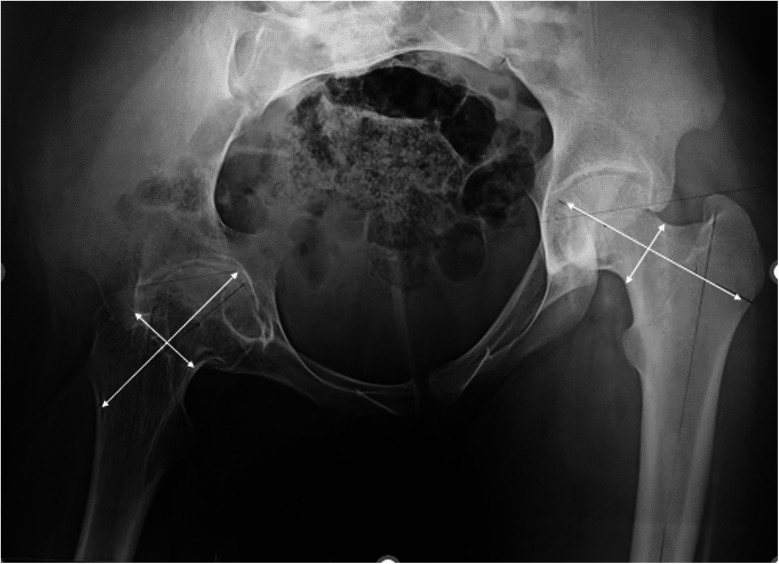
Pelvic radiograph of a 17-year-old girl with enthesitis-related arthritis JIA showing hip destruction with growth abnormalities assessed by asymmetric length and width femoral measurements.

Nineteen patients reported hip pain, with eight experiencing unilateral pain and eleven experiencing bilateral pain. Physical examination revealed limping in 11 patients and range of motion limitations on the right side in 13 patients and on the left side in 14 patients. Figures 2, 3 illustrate examples of hip involvement in two patients with JIA.

Sixteen patients underwent hip joint ultrasonography, revealing synovitis or effusion in 11 patients. Additionally, fourteen patients underwent pelvic MRI, which showed synovitis in all 14 patients, inflammatory signal abnormalities in the trochanteric area in 3 patients, and erosions in 8 patients.

### Intra-reader agreement of CARSH score

The total CARSH score, assessed by ICC, demonstrated good intra-reader agreement in R1 but low agreement in R2. Table 2 illustrates that joint space narrowing, erosion, and growth abnormalities exhibited good reliability in R1 and moderate reliability in R2. However, subchondral cysts, malalignment, and sclerosis showed poor concordance between the two observers.

**Table 2:** Intra-reader agreement of CARSH score.

CARSH score	R1			R2		
	Kappa	95% CI	*p*	Kappa	95% CI	*p*
Joint space narrowing	**0** **.** **887**	0.81–0.934	<0.001	**0** **.** **62**	0.41–0.766	<0.001
Erosion	**0** **.** **75**	0.597–0.85	<0.001	0.408	0.115–0.627	<0.001
Growth abnormalities	**0** **.** **7**	0.523–0.819	<0.001	0.424	0.17–0.625	<0.001
Subchondral cysts	0.00	−0.269 to 0.272	0.50	0.104	−0.134 to 0.347	0.201
Malalignment	0.00	−0.276 to 0.276	0.500	0.000*	−0.269 to 0.272	0.500
Sclerosis	0.086*	−0.177 to 0.344	0.265	−0.026*	−0.266 to 0.231	0.580
Total score	**0** **.** **883** *	0.801–0.932	<0.001	0.317*	0.041–0.548	0.003

CI, confidence interval; R1, reader 1; R2, reader 2.

Bold values indicate good agreement.

* ICC.

### Intra-reader agreement of the new scoring system of hip JIA

The intra-reader agreement for scoring femoral head erosion exhibited good reliability in the first observer but was poor in observer 2. Similar discordance was observed in the lecture of enlarged fovea and growth abnormalities, with high intra-reader agreement observed in only one reader. Intra-observer agreement was only fair and good in the interpretation of medial joint space narrowing. The intra-reader agreement for the new scoring system of hip JIA is summarized in Table 3.

**Table 3:** Intra-reader agreement of the new scoring system of hip JIA.

New scoring system	R1			R2		
	Kappa	95% CI	*p*	Kappa	95% CI	*p*
Erosion						
Femoral head	**0** **.** **833** *	0.719–0.903	<0.001	0.308*	−0.038 to 0.576	0.001
Femoral neck	0.237	−0.024 to 0.473	0.037	**0** **.** **631** *	0.385–0.784	<0.001
Acetabulum	0.000*	−0.269 to 0.272	0.5	−0.102*	−0.378 to 0.187	0.755
Femoral head flatting	0.290*	0.028–0.519	0.016	0.085	−0.181 to 0.347	0.269
Enlarged fovea	0.279*	0.008–0.514	0.023	**0** **.** **907** *	0.841–0.946	<0.001
Sclerosis						
Femoral head	0.475*	0.227–0.665	<0.001	−0.034*	−0.315 to 0.249	0.593
Acetabulum	0.234*	−0.035 to 0.475	0.044	**0** **.** **767**	0.617–0.862	<0.001
Joint space narrowing						
Cranial	0.238*	−0.034 to 0.048	0.044	0.352	0.086–0.573	0.006
Medial	**0** **.** **706** *	0.535–0.821	<0.001	**0** **.** **842** *	0.736–0.908	<0.001
Growth abnormalities	**0** **.** **823** *	0.707–0.896	<0.001	0.000	−0.262 to 0.270	0.5

CI, confidence interval; R1, reader 1; R2, reader 2.

Bold values indicate good agreement.

* ICC.

The intra-reader agreement for measuring the width of the femoral head showed good reliability for the first reader but was poor for the second reader (*κ*1 = 0.66 vs. *κ*2 = 0.06; *p* = 0.32). As for measuring the length of the femoral head, the reliability was slight for the first reader and good for the second one (*κ*1 = 0.23 vs. *κ*2 = 0.79; *p* < 0.001). Intra-observer agreement was absent for measuring trochanteric–femoral head length (*κ*1 = −0.068 vs. *κ*2 = −0.001; *p* = 0.5). Finally, the intra-reader agreement for scoring centrum–column–diaphysis angle showed poor reliability in the first observer, while it was slight in observer 2 (*κ*1 = 0.07 vs. *κ*2 = 0.02; *p* = 0.04).

### Training impact on readers' performances

We assessed the evolution of readers' performance by examining the difference between their first and second assessments of pelvic radiographs conducted three weeks apart using both the CARSH score and the new scoring system of the hip.

• Evolution of the reader's assessment using CARSH score

For the CARSH score, statistical analysis summarized in Table 4 showed that training has only enhanced the observers' agreement for assessing growth disorders with no improvement observed for the other items.

• Evolution of the reader's assessment using the new scoring system of hip JIA

**Table 4:** Evolution of the reader's assessment after three weeks using the CARSH score.

CARSH score	First assessment			Second assessment		
	Kappa	95% CI	*p*	Kappa	95% CI	*p*
Joint space narrowing	**0.609**	**0.382–0.763**	**<0.001**	0.560	0.339–0.723	<0.001
Erosion	0.465	0.081–0.700	<0.001	0.256	−0.019 to 0.496	0.034
Growth abnormalities	0.460	0.212–0.653	<0.001	**0** **.** **888**	**0.811–0.935**	**<0** **.** **001**
Subchondral cysts	0.028	−0.172 to 0.253	0.400	<0.000	−0.258 to 0.347	0.500
Malalignment	0.334*	0.076–0.554	0.006	−0.028*	−0.307 to 0.272	0.576
Sclerosis	0.553*	0.042–0.789	<0.001	−0.039*	−0.319 to 0.231	0.605
Total score	0.477*	−0.058 to 0.756	<0.001	0.189*	−0.083 to 0.548	0.087

CI, confidence interval.

Bold values indicate good agreement or improvement in the second assessment.

* ICC.

Regarding the second score, improvements were observed in the interpretation of sclerosis and medial joint space narrowing following training (Table 5). Concerning items with precise measurements, the readers' interpretation showed enhancements in measuring the length of the femoral head [1st assessment [(*κ* = 0.51; 95% CI (−0.239 to 0.331); *p* = 0.365) vs. 2nd assessment (*κ* = 0.793; 95% CI (0.659–0.878); *p* < 0.001)], trochanteric–femoral head length [(*κ* = 0.657; 95% CI (0.458–0.794); *p* < 0.001) vs. 2nd assessment (*κ* = 0.918; 95% CI (0.857–0.954); *p* < 0.001)] and centrum–column–diaphysis angle [(*κ* = 0.532; 95% CI (0.287–0.713); *p* < 0.001) vs. 2nd assessment (*κ* = 0.617; 95% CI (0.336–0.784); *p* < 0.001)]. However, there was no improvement observed in measuring the width of the femoral head [(*κ* = 0.612; 95% CI (0.401–0.761); *p* < 0.001) vs. 2nd assessment (*κ* = 0.345; 95% CI (0.067–0.576); *p* = 0.008)].

**Table 5:** Evolution of the reader's assessment after three weeks using the new scoring system of hip JIA.

New scoring system	First assessment			Second assessment		
	Kappa	95% CI	*p*	Kappa	95% CI	*p*
Erosion						
Femoral head	**0** **.** **608** *	**0.393–0.759**	**<0.001**	0.289*	0.011–0.527	0.022
Femoral neck	−0.058	−0.331 to 0.224	0.655	0.348*	0.083–0.569	0.006
Acetabulum	0.234*	−0.017 to 0.467	0.027	0.000	−0.250 to 0.262	0.500
Femoral head flatting	**0** **.** **876** *	**0.792–0.928**	**<0.001**	−0.016	−0.261 to 0.245	0.550
Enlarged fovea	0.075*	−0.198 to 0.341	0.296	0.173*	−0.108 to 0.428	0.113
Sclerosis						
Femoral head	−0.007	−0.281 to 0.269	0.519	**0** **.** **835** *	**0.724–0.904**	**<0** **.** **001**
Acetabulum	0.283	0.006–0.520	0.023	−0.017	−0.290 to 0.259	0.548
Joint space narrowing						
Cranial	**0.634**	**0.435–0.774**	**<0.001**	0.131	−0.144 to 0.391	0.176
Medial	0.465	0.218–0.656	<0.001	**0** **.** **615** *	**0.396–0.767**	**<0** **.** **001**
Growth abnormalities	0.188	−0.098 to 0.443	0.097	0.000	−0.262 to 0.270	0.500

CI, confidence interval.

Bold values indicate good agreement or improvement in the second assessment.

* ICC.

## Discussion

In this study, our results demonstrated moderate to good intra-reader agreement for joint space narrowing (*κ* = 0.887 vs. *κ* = 0.62; *p* < 0.001), erosion (*κ* = 0.75 vs. *κ* = 0.407; *p* < 0.001), and growth abnormalities (*κ* = 0.7 vs. *κ* = 0.424; *p* < 0.001) using the CARSH score. However, subchondral cysts, malalignment, and sclerosis showed poor concordance between the two observers. The second scoring system revealed a high ICC for only one reader in head erosion (*κ* = 0.833 vs. *κ* = 0.308; *p* < 0.001), enlarged fovea (*κ* = 0.279 vs. *κ* = 0.907; *p* < 0.05), and growth abnormalities (*κ* = 0.823 vs. *κ* = 0; *p* < 0.001, *p* = 0.5). Consequently, intra-reader agreement for femoral head measurements and centrum-column-diaphysis angle was reliable for only one reader. Training enhanced observers' agreement specifically for assessing growth disorders in the first score. Additionally, the interpretation agreement for femoral measurements improved compared to baseline.

Several studies have examined radiographic features of hip disease in children with JIA by simply describing radiographic abnormalities or structural lesions (12, 13). Using a scoring system for studying the radiographic features of hip disease in children with JIA allows standardized and quantitative assessment of radiographic joint damage. Most of our patients had enthesitis-related arthritis (ERA). This distribution can be attributed to our institution's specificities, as orthopedic colleagues referred most patients because of suspected structural joint damage, particularly in the hips. It is noteworthy that the ERA subtype exhibits the highest CARSH scores, indicating a substantial disease burden within this subgroup (14). A limited number of our patients were under bDMARDs due to resource constraints wherein access to bDMARDs is limited, making the treat to target approach challenging to implement. While bDMARDs are highly effective in reducing inflammation, there are still possibilities for structural joint damage to progress (15), especially if treatment is initiated late. Hence, radiographic scoring systems would still provide very useful role regarding long-term structural outcomes in such settings.

Joint space narrowing represented the most common radiographic abnormalities in all rheumatic diseases and was correlated with US cartilage thickness in children with JIA (16). Obviously, pediatric rheumatologists are more familiar with JSN and structural damage features than subchondral cysts and sclerosis. In contrast, during the development of the CARSH score, the pelvic radiographs of children were analyzed by the pediatric radiologists, and the agreement of the total score was good, reflecting the expertise of the radiologists (7).

Despite conducting training sessions before the study, our observations revealed lower intra-reader concordance for the second scoring system. Specifically, the intra-class correlation coefficient (ICC) was high for only one reader in scoring femoral head erosion (*κ* = 0.833 vs. *κ* = 0.308; *p* < 0.001), enlarged fovea (*κ* = 0.279 vs. *κ* = 0.907; *p* < 0.05), and growth abnormalities (*κ* = 0.823 vs. *κ* = 0; *p* < 0.001; *p* = 0.5). These findings diverged from those reported by Shelmerdine et al., where intra-reader agreement was moderate to good across all items (8). This discrepancy may be attributed to the use of electronic measurement tools in their study, which offer more accurate measurements and reduce the risk of reading errors. However, while beneficial for research purposes, the practicality of employing such software in daily clinical practice may be limited.

Consistent with the CARSH score, the interpretation of joint space narrowing (JSN) demonstrated good intra-observer agreement, underscoring the proficiency of pediatric rheumatologists in assessing this radiographic feature. However, our analysis revealed that the CARSH score tended to be more subjective in evaluating JSN compared to the newly developed scoring system. Unlike the CARSH score, which relies on subjective assessments, the newly developed scoring system offers a quantitative evaluation of the joint space at two different points (medial and cranial). This quantitative approach enhances the precision and objectivity of JSN evaluation, potentially improving the reliability and reproducibility of radiographic assessments in pediatric patients with JIA. Another limitation of the CARSH score lies in its subjective evaluation of growth abnormalities. In contrast, the new scoring system offers a more detailed assessment, providing accurate measurements in millimeters for each variable. However, despite this improvement, our analysis also revealed disagreements between observers when measuring certain variables such as the width and length of the femoral head, as well as trochanteric–femoral head length. Moreover, the intra-reader agreement for scoring centrum–column–diaphysis angle showed poor reliability. Our findings align with previous studies, which have also reported variability in the method of measuring the centrum–column–diaphysis angle, thereby limiting its comparability and reliability. Additionally, factors such as hip rotation and femoral ante- and retroversion can influence the projected angle on pelvic radiographs (17).

Despite employing the Mose circle method to assess femoral head flattening, we observed lower agreement among two readers. While the Mose circle method has been validated as a robust technique in other hip disorders in the pediatric population (18, 19), our findings suggest that it may not add precision to the evaluation of femoral head flattening. This discrepancy is consistent with the findings of Shelmerdine et al., who reported similar concordance between the Mose circle method and subjective evaluation (8).

The training sessions had a discernible impact on the readers' performances, particularly evident in the assessment of growth disorders using the CARSH score. Our observations revealed that training significantly improved the agreement among observers for identifying growth abnormalities, which were often underscored or misdiagnosed at baseline. This improvement underscores the importance of standardized interpretation using the scoring system, which emphasizes the unique characteristics of immature bones in the pediatric population. Moreover, chronic inflammation can lead to alterations in growth and bone angulation, even in the absence of erosion or bone destruction (20). Thus, familiarization with growth abnormalities is crucial for enhancing the judgment of pediatric rheumatologists when interpreting hip radiographs. For the second scoring system, our analysis revealed that training had a varying impact on readers' performances. While the interpretation of sclerosis and medial joint space narrowing (JSN) showed improvement with training for nominal qualitative items, the readers' interpretation was notably better for precise measurements of femoral head length, trochanteric–femoral head length, and the centrum–column–diaphysis angle. However, there was no discernible improvement in measuring the width of the femoral head. These findings underscore the importance of training for enhancing radiography analysis, particularly for items with precise measurements. Nevertheless, it is essential to acknowledge that such measurements are highly susceptible to pitfalls, even when performed by experienced pediatric rheumatologists. As far as know, our study was the first to use two scoring systems in the same patients with JIA and to compare their reliability and reproducibility by two pediatric rheumatologists. Our study had several limitations worth noting. Firstly, the absence of a total sum in the newly developed scoring system prevented us from correlating it directly with the established CARSH score. Secondly, despite conducting calibration workshops before the study onset, we observed a high level of disagreement between readers. We believe that incorporating more challenging cases and offering multiple calibration sessions could help overcome this discrepancy and promote the use of these scoring systems in daily practice. Additionally, further consideration should be given to the standardization of growth disturbance analysis. One approach could involve utilizing age bone-matched images as a reference atlas for assessing radiographic features, as suggested in the literature for the assessment of synovitis using ultrasound (21).

Thirdly, it's important to acknowledge that the assessment of structural damage may be insufficient for effectively monitoring children with JIA. While ultrasound and magnetic resonance imaging are preferred tools for assessing structural damage due to their accuracy and radiation-free nature (22), these resources may not be readily available in low-income countries. In such contexts, having standardized scoring systems becomes crucial for assessment. Although scoring systems standardize radiographic interpretation during clinical trials, their validity in real-world settings remains to be determined. To address this gap, further research with larger scale studies is warranted. However, we must also recognize the ethical challenges associated with obtaining pelvic radiographs in children.

In summary, our study represents the first attempt to analyze pediatric hip radiograph variables associated with JIA using two scoring systems and assess their applicability in daily clinical practice, as well as their reliability when interpreted by pediatric rheumatologists. While the CARSH reference score offered ease of use and reliability, it remained subject to subjective interpretation. Conversely, the novel score provided greater precision but lacked a total sum and was more time-consuming. It became evident that the reliability of these tools was compromised without electronic measurements, underscoring the need for additional training among pediatric rheumatologists before incorporating these scoring systems into routine hip monitoring practices.

## Data Availability

The raw data supporting the conclusions of this article will be made available by the authors, without undue reservation.

## References

[1] Houghton KM Macdonald HM McKay HA Guzman J Duffy C Tucker L Feasibility and safety of a 6-month exercise program to increase bone and muscle strength in children with juvenile idiopathic arthritis. Pediatr Rheumatol Online J. (2018) 16(1):67. 10.1186/s12969-018-0283-4 30348221 PMC6198360

[2] Shelmerdine SC Di Paolo PL Tanturri de Horatio L Malattia C Magni-Manzoni S Rosendahl K. Imaging of the hip in juvenile idiopathic arthritis. Pediatr Radiol. (2018) 48(6):811–7. 10.1007/s00247-017-4022-7 29766251

[3] Naveen R Mohindra N Jain N Majumder S Aggarwal A. Hip involvement in children with enthesitis-related arthritis (ERA) is associated with poor outcomes in adulthood. Clin Rheumatol. (2021) 40(11):4619–27. 10.1007/s10067-021-05807-3 34169374 PMC8225398

[4] Hamdi W Ferjani H Carlomagno R Dusser P Echaubard S Belot A Factors associated with poor prognosis of hip arthritis in juvenile idiopathic arthritis: data from the JIR cohort. Musculoskeletal Care. (2023) 21(3):806–14. 10.1002/msc.1755 36896923

[5] Onel KB Horton DB Lovell DJ Shenoi S Cuello CA Angeles-Han ST 2021 American college of rheumatology guideline for the treatment of juvenile idiopathic arthritis: therapeutic approaches for oligoarthritis, temporomandibular joint arthritis, and systemic juvenile idiopathic arthritis. Arthritis Rheumatol. (2022) 74(4):553–69. 10.1002/art.42037 35233993 PMC10161784

[6] Ringold S Angeles-Han ST Beukelman T Lovell D Cuello CA Becker ML 2019 American college of rheumatology/arthritis foundation guideline for the treatment of juvenile idiopathic arthritis: therapeutic approaches for non-systemic polyarthritis, sacroiliitis, and enthesitis. Arthritis Care Res. (2019) 71(6):717–34. 10.1002/acr.23870 PMC6561125 31021516

[7] Bertamino M Rossi F Pistorio A Lucigrai G Valle M Viola S Development and initial validation of a radiographic scoring system for the hip in juvenile idiopathic arthritis. J Rheumatol. (2010) 37(2):432–9. 10.3899/jrheum.090691 20032107

[8] Shelmerdine SC Di Paolo PL Rieter JFMM Malattia C Tanturri de Horatio L Rosendahl K. A novel radiographic scoring system for growth abnormalities and structural change in children with juvenile idiopathic arthritis of the hip. Pediatr Radiol. (2018) 48(8):1086–95. 10.1007/s00247-018-4136-6 29717335 PMC6061460

[9] Krumrey-Langkammerer M Häfner R. Evaluation of the ILAR criteria for juvenile idiopathic arthritis. J Rheumatol. (2001) 28(11):2544–7. 11708431

[10] Consolaro A Ruperto N Bazso A Pistorio A Magni-Manzoni S Filocamo G Development and validation of a composite disease activity score for juvenile idiopathic arthritis. Arthritis Rheum. (2009) 61(5):658–66. 10.1002/art.24516 19405003

[11] Hallgren KA. Computing inter-rater reliability for observational data: an overview and tutorial. Tutor Quant Methods Psychol. (2012) 8(1):23–34. 10.20982/tqmp.08.1.p023 22833776 PMC3402032

[12] Van Rossum MAJ Zwinderman AH Boers M Dijkmans BAC Van Soesbergen RM Fiselier TJW Radiologic features in juvenile idiopathic arthritis: a first step in the development of a standardized assessment method. Arthritis Rheum. (2003) 48(2):507–15. 10.1002/art.10783 12571861

[13] Selvaag AM FlatØ B Dale K Lien G Vinje O Smerdel-Ramoya A Radiographic and clinical outcome in early juvenile rheumatoid arthritis and juvenile spondyloarthropathy: a 3-year prospective study. J Rheumatol. (2006) 33(7):1382–91. 16758503

[14] Ferjani HL Dhia SB Nessib DB Dghaies A Kaffel D Maatallah K The childhood arthritis radiographic score of the hip: the proposal cut-off value using cluster analysis. Clin Rheumatol. (2024) 43(1):465–72. 10.1007/s10067-023-06749-8 37635192

[15] Aoki C Inaba Y Choe H Kaneko U Hara R Miyamae T Discrepancy between clinical and radiological responses to tocilizumab treatment in patients with systemic-onset juvenile idiopathic arthritis. J Rheumatol. (2014) 41(6):1171–7. 10.3899/jrheum.130924 24786929

[16] Pradsgaard DØ Hørlyck A Spannow AH Heuck C Herlin T. A comparison of radiographic joint space width measurements versus ultrasonographic assessment of cartilage thickness in children with juvenile idiopathic arthritis. J Rheumatol. (2019) 46(3):301–8. 10.3899/jrheum.170571 30442828

[17] Boese CK Dargel J Oppermann J Eysel P Scheyerer MJ Bredow J The femoral neck-shaft angle on plain radiographs: a systematic review. Skeletal Radiol. (2016) 45(1):19–28. 10.1007/s00256-015-2236-z 26305058

[18] Clohisy JC Carlisle JC Trousdale R Kim YJ Beaule PE Morgan P Radiographic evaluation of the hip has limited reliability. Clin Orthop Relat Res. (2009) 467(3):666–75. 10.1007/s11999-008-0626-4 19048356 PMC2635468

[19] Cuomo AV Fedorak GT Moseley CF. A practical approach to determining the center of the femoral head in subluxated and dislocated hips. J Pediatr Orthop. (2015) 35(6):556–60. 10.1097/BPO.0000000000000281 26090988

[20] Bechtold S Simon D. Growth abnormalities in children and adolescents with juvenile idiopathic arthritis. Rheumatol Int. (2014) 34(11):1483–8. 10.1007/s00296-014-3022-2 24760485

[21] Sande NK Bøyesen P Aga AB Hammer HB Flatø B Roth J Development and reliability of a novel ultrasonographic joint-specific scoring system for synovitis with reference atlas for patients with juvenile idiopathic arthritis. RMD Open. (2021) 7(2):e001581. 10.1136/rmdopen-2021-001581 33883255 PMC8061832

[22] Porter-Young FM Offiah AC Broadley P Lang I McMahon AM Howsley P Inter- and intra-observer reliability of contrast-enhanced magnetic resonance imaging parameters in children with suspected juvenile idiopathic arthritis of the hip. Pediatr Radiol. (2018) 48(13):1891–900. 10.1007/s00247-018-4216-7 30076429

